# Stimulation of *Acidimicrobium* sp. Strain A6 Biodegradation of PFOS in AFFF-Impacted Sediment Columns Using PAA-Coated Goethite

**DOI:** 10.1021/acsestengg.6c00272

**Published:** 2026-06-30

**Authors:** Matthew W. Sima, Shan Huang, Peter R. Jaffé

**Affiliations:** Department of Civil and Environmental Engineering, School of Engineering and Applied Science, 6740 Princeton University, Princeton, New Jersey 08544, United States

**Keywords:** Biological degradation, PFAS defluorination, Feammox, *Acidimicrobium* sp. strain A6, Soil treatment

## Abstract

PFAS biodegradation at aqueous film-forming foam (AFFF)-impacted sites may be achieved by leveraging the native biogeochemical properties of the soil. Previous studies have shown that *Acidimicrobium* sp. strain A6 (A6), an ammonium-oxidizing iron reducer capable of Feammox, can be biostimulated to degrade PFOA and PFOS in sediment slurries under controlled laboratory incubations. However, implementing this biostimulation in the field requires distributing Fe­(III) throughout the sediment. To do so, we lowered the zeta potential of goethite by coating it with polyacrylic acid (PAA) and conducted column experiments under different iron levels across three experimental setups: AFFF-contaminated sediments, ^12^C_8_–PFOS-spiked AFFF-contaminated sediments, and ^13^C_8_–PFOS-spiked AFFF-contaminated sediments. Results show that PAA-coated goethite treatment decreased PFOS mobilization and enhanced A6 activity, as demonstrated by increased Feammox activity and increased A6 counts. This biostimulation promoted PFOS degradation, as evidenced by the production of F^–^ and shorter carbon-chain intermediates. Greater stimulation was observed in soils with high initial Fe­(III) content, indicating the importance of initial soil geochemical conditions for A6 activity. This stimulation of A6 in natural soils via the addition of PAA-coated goethite is highly promising for environmental remediation of PFAS-contaminated sediments and warrants further optimization and testing under field-relevant conditions.

## Graphical abstract

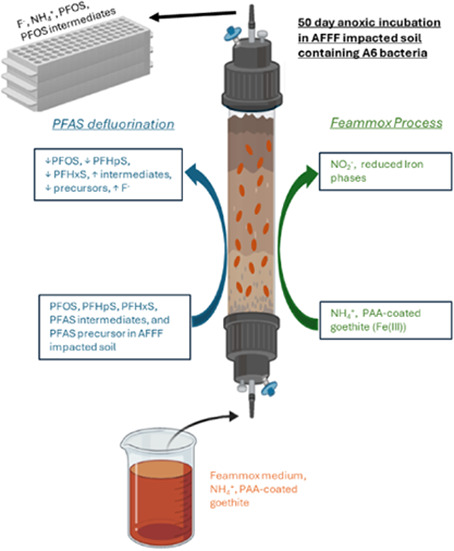

## 1 Introduction

The use of aqueous film-forming foam (AFFF) has resulted in extensive PFAS contamination of soil and groundwater.^1,2^ Since the early 1960s, AFFF formulations have contained perfluorosulfonic acids (PFSAs), such as PFOS, and their precursors as primary firefighting agents, in addition to various other proprietary PFAS mixtures.^3^ The heat resistance and chemical stability of PFOS enable it to act as a blanket, smothering oil fires and preventing oxygen from reaching flames. This same stability impedes the breakdown of PFOS, allowing it to infiltrate the surrounding soil and form persistent PFAS plumes as they disperse.

Once in the environment, perfluorinated precursors and polyfluorinated compounds in AFFF can be transformed into their more stable perfluorinated degradation products, specifically perfluorocarboxylic acids (PFCA) and perfluorosulfonic acids (PFSA).^1,4,5^ Reports have indicated that PFAS precursors account for 41–100% of the total molar PFAS concentration in historical electrochemical fluorination (ECF)- and fluorotelomer (FT)-based AFFF formulations.^6^ However, once deployed, the proportion of precursors can decrease to 23–28% of the total PFAS in groundwater and soil samples, accompanied by a corresponding increase in the fraction of PFCAs and PFSAs relative to the initial AFFF formulation.^7^ Once they have been transformed into perfluorinated compounds, these PFAS become stable in the environment and become exceedingly difficult to degrade, thus resulting in their designation as “Forever Chemicals”.^8–11^

Currently, neither physical nor chemical remediation methods can fully remove either PFCAs or PFSAs from contaminated soils. Physical remediation approaches such as biochar amendment can immobilize PFAS; however, this approach merely produces secondary PFAS-laden waste, with limited understanding of subsequent desorption or transformation as biochar is subjected to varying soil conditions.^12,13^ Chemical remediation requires highly aggressive conditions, such as elevated temperatures and corrosive reagents, which are detrimental to native soil microbial communities.^13^ Alternatively, bioremediation, despite being presumed ineffective for remediating perfluorinated compounds, may represent a key strategy for the remediation of environmental PFAS.

While many studies have shown the degradation of polyfluoroalkyl substances, as supported by the buildup of perfluorinated intermediates and F^–^ ions, very few microorganisms have been reported to mineralize perfluorinated compounds. Yi et al. (2016) reported the removal of 48.1% PFOA by *Pseudomonas parafulva* strain YAB-1 but without reporting the presence of degradation products.^14^ Likewise, Kwon et al. (2014) reported a decrease in PFOS by *Pseudomonas aeruginosa* strain HJ4, as supported by increases in PFHxS and PFBS.^15^ However, none of these studies are supported by F^–^ release, and no fluorine mass balances were performed to confirm PFOS mineralization.

Huang and Jaffe (2018) isolated a bacterium, *Acidimicrobium* sp. strain A6 (hereafter referred to as A6), a slow-growing autotroph (doubling time: 8 to 10 days) that oxidizes ammonium to nitrite while reducing ferric iron in a process known as Feammox.^16^ A6 has been shown to be relatively common in acidic, iron-rich soils, and its activity can be stimulated in sediment incubations treated with Fe­(III), NH_4_^+^, and a Feammox growth medium.^17^ A6 has also been shown to defluorinate PFAS by coupling it with the Feammox process.^18–24^ Degradation of PFOA was supported by the detection of perfluorinated intermediates, namely perfluoroheptanoic acid (PFHpA), perfluorohexanoic acid (PFHxA), perfluoropentanoic acid (PFPeA), and perfluorobutanoic acid (PFBA), and F^–^. A fluorine mass balance was conducted with high F recovery.^18^ Furthermore, the genome of A6 includes sequences that encode several dehalogenases, one of which, a novel reductive dehalogenase (*rdhA*) showed a strong correlation between its expression and F^–^ produced during incubations with A6 in which PFAS defluorination was observed.^21^

As mentioned, A6 bacteria require specific geochemical conditions to undergo the Feammox process and PFAS defluorination, including an anoxic environment, low pH, high ferric iron as an electron acceptor, NH_4_^+^ as an electron donor, and synergistic heterotrophic bacteria. A recent study by Huang et al. (2024b) showed that naturally occurring A6 in AFFF-impacted sediments that meet these conditions was able to degrade PFOA and PFOS in laboratory biostimulated sediment slurry incubations, as supported by PFOA and PFOS degradation products and F^–^ production.^20^ While these laboratory experiments have shown the ability of A6 to degrade PFAS in sediment slurry environments treated with ferric iron and NH_4_^+^, the question remains whether it is feasible to deliver the requisite solid ferric iron phase to stimulate naturally occurring A6 and PFAS defluorination in AFFF-contaminated sites within a soil matrix exhibiting appropriate geochemical conditions.

While providing NH_4_^+^ to a soil/sediment matrix is relatively straightforward, supplying a solid ferric iron phase is considerably more challenging, especially given the positive charge of ferric iron and the typical negative charge of the soil. Park et al. (2023) showed that coating of ferric iron with polyacrylic acids (PAA) not only lowers the zeta potential of the positively charged ferric iron, thereby facilitating improved transport through the negatively charged soil matrix,^22,25^ but also enhances transport of electrons to the surface of the ferric iron, thereby increasing both the rate of Feammox activity and PFAS defluorination. This PAA-coating approach is applicable to multiple ferric iron mineral phases, including ferrihydrite and the more readily available goethite.^22^

Therefore, the objective of the work described here was to test the effectiveness of applying PAA-coated ferric iron to sediment columns to stimulate A6-mediated PFAS defluorination based on Huang et al. (2024b) and Park et al. (2023).^20,22^ Two sediments from DoD sites with a history of AFFF applications and which contained naturally occurring A6 were treated with PAA-coated goethite.^22^ LC–MS was used to measure PFAS and their degradation products in the effluent and sediment samples. Microbial community analysis and qPCR were conducted to track A6 numbers and investigate the effect of PAA-coated goethite on the microbial community.

## 2 Materials and Methods

### 2.1 Sediment Physiochemical Properties

Iron-rich and acidic AFFF-impacted sediments from two different DoD sites located in the Northern Atlantic Coastal Plain were used for these column experiments. These sediments had previously been tested for the presence of A6 and correspond to Sediment #2 (Sed-2) and Sediment #3 (Sed-3) from Huang et al. (2024b). Sed-2 had three times higher ferric iron content (61.2 mg/kg vs 20.3 mg/kg), 5-fold higher TOC content (117.8 mg/kg vs 23.5 mg/kg), and lower pH than Sed-3 (6.04 vs 6.57). Initial PFAS concentrations were tested commercially by the Canadian branch of Société Générale de Surveillance (SGS) using EPA Method 1633 as shown in Table S2 and verified in-house via extraction with methanol and using an LC–MS. Initial PFOS concentrations were 1,370 ppb (SGS measurement) and 1,105 ± 389 ppb (in-house measurement) for Sed-2, and 2,590 ppb (SGS measurement) and 2,564 ± 114 ppb (in-house measurement) for Sed-3. Further information on the sediment properties and chemical composition can be found in Table S1 and in Huang et al. (2024b).^20^

Three sets of contaminated sediment column experiments were conducted: 1) columns without soil modification to investigate degradation of existing PFAS, a list of which is shown in Table S2; 2) columns augmented with ^12^C_8_–PFOS to get a better F mass balance; and 3) columns augmented with ^13^C_8_–PFOS (all 8 carbons labeled with ^13^C) to better track PFOS degradation and the production of degradation intermediates without the confounding effects of precursor degradation. For set 2, 1000 ± 1 g of each sediment type was thoroughly mixed with 40.5 ± 3 mg of PFOS in DI water and air-dried in the fume hood for 24 h before use. For set 3, the sediments were spiked with ^13^C–PFOS by mixing 1 mg of ^13^C_8_–PFOS per 250 mg of sediment before loading the sediments into the columns. A lower concentration of ^13^C_8_–PFOS was used in set 3 due to cost constraints and the absence of a mass balance requirement.

### 2.2 Inflow Solution (Influent)

For each set, columns were split into three different operating conditions: **C1 (DIW)**, deionized water (DIW) control; **C2 (NH**_**4**_^**+**^ **treatment condition)**, NH_4_^+^ amended condition with bacterial nutrient medium (adopted from Huang et al., 2024b and listed in SI); and **C3 (Fe­(III) treatment condition)**, NH_4_^+^ amended condition with bacterial nutrient medium and 6k-PAA-coated goethite. All inflow solutions were adjusted to pH 4.5 by using HCl.

The 6k-PAA was prepared by dissolving 2.6 g of sodium PAA (molecular weight 6000, Polysciences, white powder) in 1 L of DIW. A 10x stock solution was prepared by adding 300 mL of stock 6k-PAA to 1500 mg of goethite (M = 5g/mol; CAS no.: 20344-49-4; Sigma-Aldrich; powder) to a final volume of 1 L. This suspension was then homogenized and added to a nutrient medium accordingly, to obtain a final medium consisting of 65 mg/L NH_4_^+^, 180 mg of PAA-coated goethite, and the same microbial nutrient composition as in C2.

### 2.3 Sediment Column Setup

Each column (borosilicate glass, 2.5 cm ID, 30 cm long) was filled with approximately 250 g of homogenized sediment from its respective site, and the columns were gently tamped to ensure uniform packing of the sediment. Larger solid particles and other obstructions, such as stones and roots, were removed prior to loading the sediment into the columns. Paper filters were placed at the ends of the columns to prevent sediment loss during operation. The oven-dry weight of the sediment in the column was 227.28 ± 2.2 g and 209.1 ± 1.1 g for Sed-3 and Sed-2, respectively. The Sed-3 columns had a bulk density of 1.54 g/cm^3^, a porosity of 0.22 ± 0.009, and a pore volume of 25.9 ± 1.0 mL, while the Sed-2 columns had a bulk density of 1.42 g/cm^3^ and a porosity of 0.19 ± 0.002 with a pore volume of 22.2 ± 0.2 mL.^26–28^ Each column was purged upward with CO_2_ to ensure better water saturation of the sediment.

The columns were operated in an upward-flow mode at 0.33 mL/h for Sed-2 and 3 mL/h for Sed-3. The flow rate for Sed-2 was lower despite an equivalent pump setting, as the higher clay content of Sed-2 substantially restricted flow through the column relative to the sandy sediment texture of Sed-3.

Twelve columns were operated in parallel, six for each sediment, with two replicates for each of the three operating conditions mentioned above. The respective influent for each condition was placed in a common homogenized solution reservoir, thereby standardizing it across sediment types and replicates. The outflow from the columns (effluent) was collected using fraction collectors and analyzed using ion chromatography for NH_4_^+^ and F^–^ and using liquid chromatography–mass spectrometry for PFAS. pH was analyzed directly from the outflow using pH paper and confirmed using pH electrodes.

For each sediment, columns were operated until PAA-coated goethite breakthrough was observed in the respective C3 column to ensure that each column was uniformly conditioned with its respective influent solution. Since typical methods of ferric iron analysis were unable to measure total Fe­(III) from goethite, and the coating of the goethite by PAA made Fe­(III) analysis even more difficult,^29^ the concentration of PAA-coated goethite in the effluents was determined via UV–vis light attenuation. Although the initial few pore volumes of Sed-3 contained significant amounts of fine particles, after 3 pore volumes these fine particles were negligible, and samples could be analyzed for goethite breakthrough using a UV–vis for three different wavelengths: 330, 435, and 836 nm (Figure S1). Breakthrough of the PAA-coated goethite in these columns occurred at ∼10 pore volumes.

All columns were run for approximately 10 pore volumes, despite C1 and C2 reaching breakthrough before C3. Once breakthrough of the PAA-coated goethite was achieved for all columns, pumping was ceased, inflow and outflow valves were closed, and the sediments in each column were allowed to incubate at room temperature for 50 days prior to resuming flow. As shown by a five-day biochemical oxygen demand (BOD_5_) test described in the SI, the soil organic matter (SOM) content was sufficient to support microbial respiration, leading to oxygen depletion after 5 days.

Once pumping was resumed, the effluent from each column was collected for an additional 5 pore volumes in a fraction collector in 4 mL increments and was again tested for pH, NH_4_^+^, F^–^, and PFAS. The columns were then disassembled and split into three sections: top (20–30 cm), middle (10–20 cm), and bottom (0–10 cm). Sediments from each section were homogenized and analyzed for PFAS concentration, A6 bacterial counts, and microbial community analysis.

### 2.4 Non-PFOS-Spiked Impacted-Sediment Column Setup

Experiment 1 was conducted on sediments without modification (Tables S1 and S2) according to the column setup described above. After the 50-day incubation and the subsequent collection of effluent after pumping was restarted, sediment samples from each of the three sections (top, middle, and bottom) were collected, homogenized, and sent to SGS Shanghai for microbial community analysis and A6 quantification and to SGS Canada for PFAS quantification using EPA Method 1633.

A fluorine mass balance was not conducted for this experiment since previous experiments have shown that F^–^ can bind to ferric iron when it is reoxidized from ferrous iron at higher pH,^30^ resulting in a poor mass balance at these ambient F^–^ concentrations. Hence, F^–^ was used here only as a qualitative indicator of PFAS defluorination.

### 2.5 ^12^C_8_–PFOS-Spiked Sediment Column Experiment Setup

In the nonspiked sediment experiments, PFOS represented the predominant detected PFAS, constituting 46% in Sed-2 and 42% in Sed-3; however, both sediments also contained varying initial concentrations of other PFOS precursors and transformation intermediates. Therefore, to better constrain the fluorine source and mitigate uncertainty arising from PFOS precursors, PFOS-spiked sediment column experiments were conducted at 40 mg/kg to facilitate a more rigorous fluorine mass balance. After the results of the nonspiked experiments showed little A6 activity in the C1 DIW columns, only C2 and C3 columns were run in duplicate for the PFOS-spiked column experiments.

The Sed-2 and Sed-3 sediments were spiked with PFOS before loading into the columns. 1000 ± 1 g of each sediment type was thoroughly mixed with 40.5 ± 3 mg of PFOS in DI water and air-dried in the fume hood for 24 h. The sediment was then loaded into the columns, and the entire column was weighed. The Sed-2 columns contained 236.4 ± 1.5 g of air-dried sediment each, and the Sed-3 columns contained 234.1 ± 4.2 g of air-dried sediment each. The remaining sediment was used for PFAS methanol extraction, followed by LC–MS analysis. Total extracted PFOS concentrations in the sediment were 42.7 ± 2.7 mg/kg for Sed-2 and 48.8 ± 10.2 mg/kg for Sed-3. The total mass of PFOS in each Sed-2 column was approximately 10.3 ± 1.6 mg, while the total mass of PFOS in each Sed-3 column was 11.5 ± 2.4 mg.

Each column was run as described above, and a fluorine mass balance was conducted by summing up the free F^–^ and the fluorine present in PFAS compounds measured for day 0 effluent, day 50 effluent, and sediment extracts after 50 days of operation.

### 2.6 ^13^C_8_–PFOS-Spiked Sediment Column Experiment Setup

For this experiment, sediments were spiked with 1 mg of ^13^C_8_–PFOS (all 8 carbons labeled) per 250 mg of sediment before loading the sediments into the columns to allow for a more accurate testing of the production of PFOS intermediates. The experimental setup was identical to that of the PFOS-spiked sediment columns, with only C2 and C3 columns run in duplicate. Each column was run according to the column procedure above for 50 days, and the effluent samples were analyzed for Feammox activity, ^13^C_8_–PFOS, and ^13^C-labeled intermediates. The columns were then split into thirds (top, middle, and bottom), and sediments were analyzed for A6 numbers, ^13^C_8_–PFOS, and ^13^C-labeled intermediates.

### 2.7 Chemical Analyses

The chemical analyses were adopted from Huang et al. (2019) and Huang et al. (2024b) and presented in the SI. ^13^C_8_ PFOS and its intermediates were analyzed using the LCMS-2050 Single Quadrupole Mass Spectrometer (Shimadzu) in accordance with EPA Method 537.1.^18,20,31,32^ The column used during analysis was a Shim-pak Velox C18 column (I.D. 2.1 mm, length 50 mm, particle size 2.7 μm) from Shimadzu at a flow rate of 0.4 mg/min at 40 °C. The ^13^C intermediates were initially detected by running a series of *m*/*z* ratios (defined as the target *m*/*z* ratio ± 1 ([M–H]–1)) for the specific target intermediate to identify significant chromatographic peaks. To simulate the additional neutron in the ^13^C, compared to the more common ^12^C, a H was added to the chemical formulation to increase the mass by 1 without affecting the charge. These intermediates were then compared to ^13^C standards from Wellington Laboratories Inc. and Cambridge Isotope Laboratories Inc. to identify the correct retention time and intermediate products.

### 2.8 Microbial and Genomic Analyses

Microbial and genomic analyses were conducted in triplicate with DNA samples extracted using the FastDNA spin kit for soil (MP Biomedicals), and the concentration and quality were checked using a Qubit 2.0 Fluorometer (Thermo Scientific). qPCR was conducted using the One Step SYBR PrimeScript RT-PCR Kit II (TaKaRa, Japan) following the manufacturer’s instructions on a 96-well StepOnePlus Real-Time PCR System (Applied Biosystems, CA, USA). Methods were conducted using the protocols described in Huang and Jaffé (2018), expanded later in Huang et al. (2024b), and further details are listed in the SI.^16,20^

## 3 Results and Discussion

### 3.1 Nonspiked Sediment Column Results

#### 3.1.1 Feammox Activity after Stimulation with PAA-Coated Goethite

Feammox activity was determined based on changes in the NH_4_^+^ concentrations and pH, with a greater decrease in NH_4_^+^ and greater increase in pH indicative of enhanced Feammox activity, as shown in the equation below.

1 $$3{\mathrm{Fe}}_{2}O_{3}•0.5H_{2}O+10H^{+}+{{\mathrm{NH}}_{4}}^{+}\to 6{\mathrm{Fe}}^{2+}+8.5H_{2}O+{{\mathrm{NO}}_{2}}^{-}$$

Since the traditional ferrozine method of measuring the Fe­(II)/Fe­(III) ratio is inadequate for PAA-coated goethite,^22^ only the decrease in NH_4_^+^ and increase in pH were used as Feammox indicators.

As shown in Figure 1, after 50 days of incubation, the C3 (Fe­(III) + NH_4_^+^) columns resulted in greater NH_4_^+^ removal compared to the C2 (NH_4_^+^) condition. The NH_4_^+^ concentration in Sed-2 C3 (Fe­(III) + NH_4_^+^ treatment) decreased from 65 ± 1 mg/L to 50.8 ± 4.3 mg/L, while the NH_4_^+^ concentration in Sed-3 C3 (Fe­(III) + NH_4_^+^ treatment) decreased from 65 ± 1 mg/L to 60.8 ± 2.4 mg/L. The Sed-2 columns had greater NH_4_^+^ removal than the Sed-3 columns, regardless of treatment.

**Figure 1:**
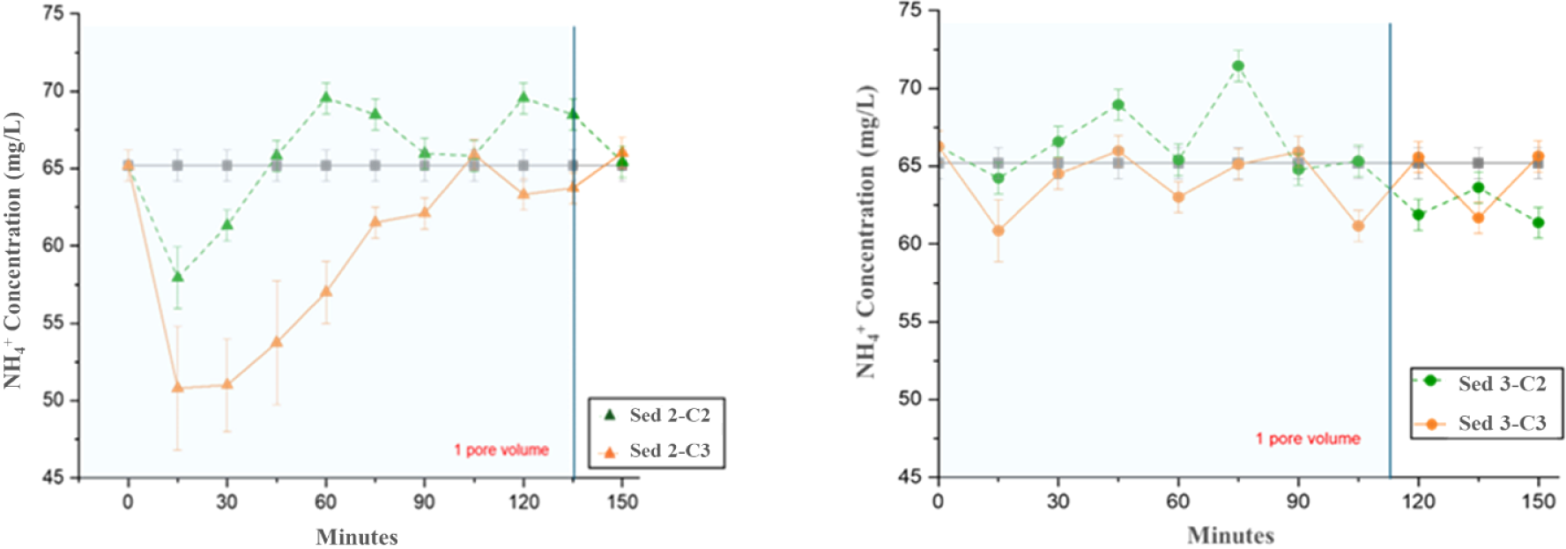
NH_4_^+^ removal after 50 days (C2 is the NH_4_^+^ no-iron treatment; C3 is the Fe­(III) and NH_4_^+^ experimental treatment). C1 (DIW treatment) was not included because no NH_4_^+^ was amended to those columns. The gray line at 65 mg/L represents the NH_4_^+^ concentration in the influent used to flush the columns after 50 days. These graphs compare C2 and C3 for Sediment 2 (left) and Sediment 3 (right). The results show greater NH_4_^+^ removal in Sed 2 than in Sed 3, and in C3 than in C2.

All columns started at pH 4.5, and the increase in pH was greater in the Fe­(III)-treated columns: Sed-2 effluents had a greater increase in pH to 6.5 and 6 for C3 (Fe­(III) + NH_4_^+^ treatment) and C2 (NH_4_^+^ treatment), respectively, compared to Sed-3, which showed an increase to 6 and 5, respectively, for C3 (Fe­(III) + NH_4_^+^ treatment) and C2 (NH_4_^+^ treatment). These results indicate higher Feammox activity in C3 (Fe­(III) + NH_4_^+^ treatment) compared with C2 (NH_4_^+^ treatment) and higher Feammox activity in Sed-2 compared with Sed-3.

#### 3.1.2 PFOS Concentrations and Production of F^–^

After 50 days, F^–^ production in the effluent was measured as a qualitative indicator of PFAS defluorination. Both sediments showed the production of F^–^, as shown in Figure 2; however, the addition of PAA-coated goethite affected the recovery of F^–^ from the sediment matrix. This may be due to the increase in pH during the experiment, which has been shown to limit the amount of extractable F^–^ in Fe­(III)-rich systems. As mentioned above, previous experiments have shown that in incubations when the final pH was >6, only a poor fluorine balance is achieved^33^ due to the possible formation of several stable fluoro complexes of Fe­(III).^30^

**Figure 2:**
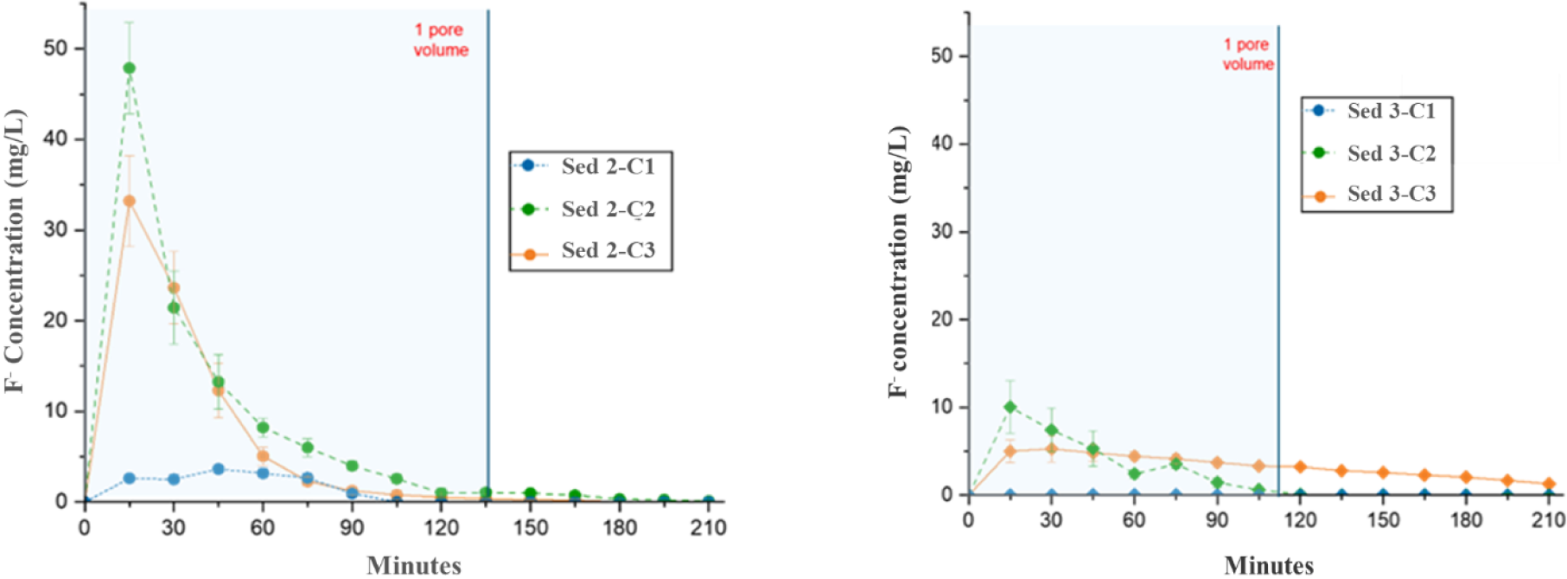
F^–^ production between Sed-2 and Sed-3 C1, C2, and C3 columns (C1 is the DIW control; C2 is the NH_4_^+^ no-iron treatment; C3 is the Fe­(III) and NH_4_^+^ experimental treatment). These graphs compare C2 and C3 for Sediment 2 (left) and Sediment 3 (right). The results show greater F^–^ production in Sed 2 than in Sed 3, and in C3 than in C2 or C1.

After flushing the columns with 5 pore volumes of DIW (C1) or respective media (C2 and C3), they were disassembled and split into three sections (top, middle, and bottom). Each section was homogenized and analyzed for the PFAS. The results show that the PFOS concentration by column height exhibited a greater variation between treatments, with C1 (DIW treatment) having a greater PFOS concentration closer to the outflow of the column (top), while C2 (NH_4_^+^ treatment) and C3 (Fe­(III) + NH_4_^+^ treatment) had a greater PFOS concentration closer to the inflow of the column (bottom). The higher PFOS near the outflow for C1 (DIW) may be due to PFOS mobilization from the bottom of the column to the top during the pumping process, which is supported by the lower partitioning in the presence of DI (Table S3). In comparison, the decrease in PFOS in C2 and C3 closer to the outflow of the column could be due to lower DO in the medium, as it is consumed by microbial respiration, leading to greater Feammox activity and PFOS defluorination in the anoxic sediment closer to the outflow.

When the three column sections were averaged, Sed-3 columns, with lower Feammox activity and lower initial Fe­(III) concentration, did not exhibit significant differences in PFOS concentrations between C1, C2, and C3 (1493.3 ± 278.1 μg/L, 1554.3 ± 67.3 μg/L, and 1247.9 ± 165.0 μg/L for C1, C2, and C3, respectively), whereas Sed-2 columns, with greater NH_4_^+^ oxidation, exhibited significant differences between the three treatments (1105.1 ± 389.7 μg/L, 652.7 ± 172.9 μg/L, and 459.7 ± 48.7 μg/L for C1, C2, and C3, respectively). This suggests that the elevated native Fe­(III) in Sed-2 supports a more robust A6 community, which in turn responds more favorably to biostimulation and A6 enrichment upon addition of PAA-coated goethite, as illustrated in Figure 3.

**Figure 3:**
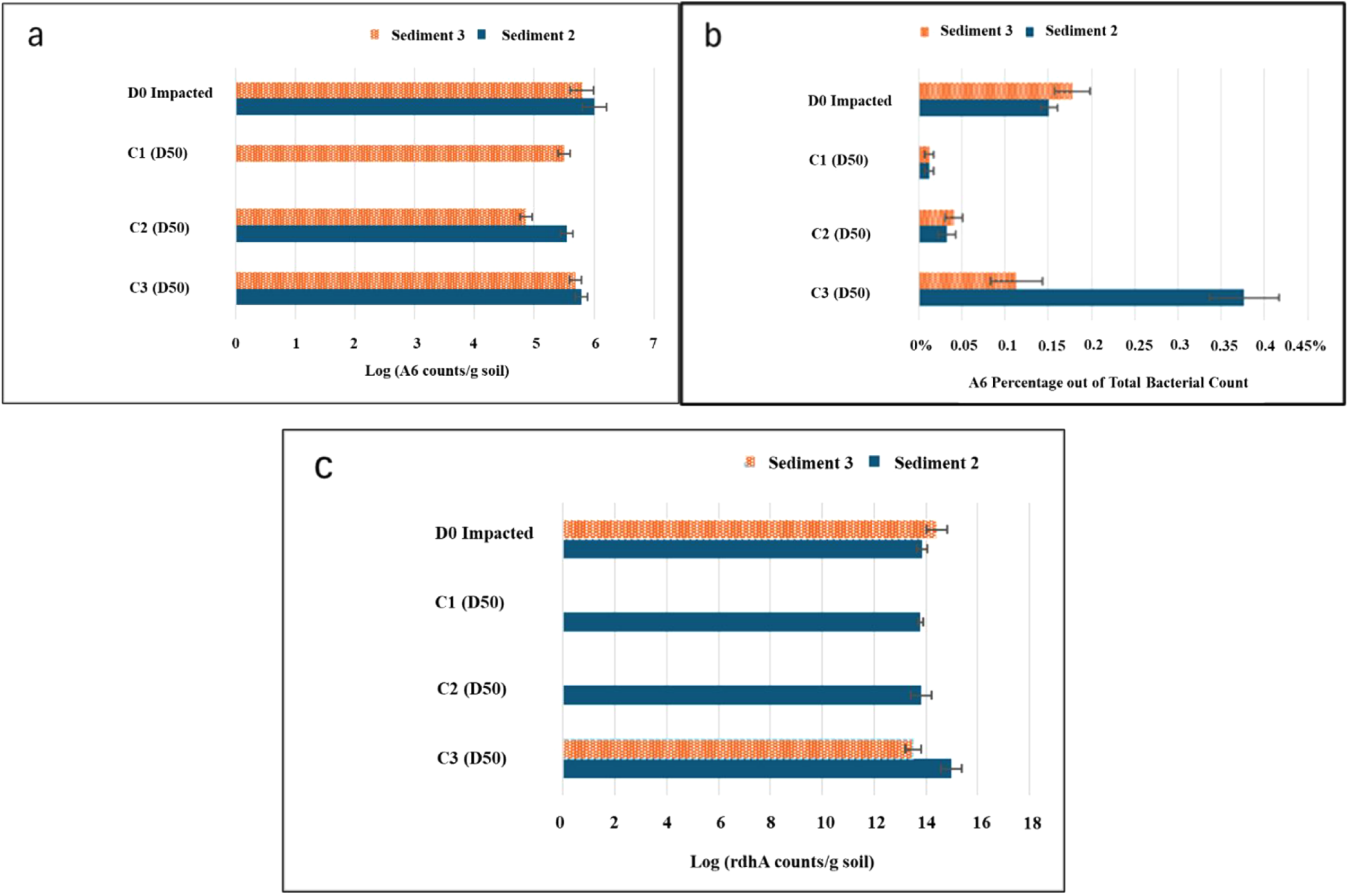
When comparing the Log (A6 counts/g soil) across the three conditions, it is evident that flushing had an adverse effect on A6 counts across all conditions. While A6 numbers for C3 reached levels comparable to the D0 numbers after 50 days, the A6 percentage of the total bacterial count showed that the A6 percentage in Sed-2 C3 increased over 2-fold. A similar trend is seen in rdhA counts, which increased over 50 days in C3 compared to C1 or C2.

#### 3.1.3 Effect of PAA-Coated Goethite on A6 Numbers

Given the long doubling time of A6 (approximately 10–15 days), A6 cell counts are not expected to increase by orders of magnitude over the 50-day stimulation period; however, results show that the addition of PAA-coated goethite resulted in a significant shift in microbial community with a positive effect on the A6 numbers, A6%, *rdh*A copy counts, and microbial diversity. Figure 3 shows the distribution of A6 and *rdh*A across conditions and between sediments. Figure 3a shows the A6 numbers in log (counts/g sediment), and Figure 3b shows the percentage of A6 out of the total bacterial count. On average, flushing had a negative impact on A6 counts; even so, C3 (Fe­(III) + NH_4_^+^ treatment) showed the greatest A6 counts between the conditions and approached the initial pretreatment values following the 50-day incubation. When converted to A6%, results showed the greatest increase in the C3 (Fe­(III) + NH_4_^+^ treatment) columns compared to either the C1 or C2 columns. Similar to the A6 numbers, the initial flushing of the columns did adversely impact the A6%, however, for the Sed-2 C3 column, the A6% more than doubled after the 50-day incubation.

Given the low A6 counts, the reductive dehalogenase (*rdhA*) gene counts may also serve as an indicator of A6’s defluorination potential. Figure 3C shows that for Sed-2 C3, *rdhA* gene counts tripled over 50 days in the presence of PAA-coated goethite. In contrast, the slower defluorination rate of Sed-3 C3 compared to Sed-2 C3 can be attributed to lower *rdhA* gene counts on day 50. Therefore, *rdhA* gene counts and A6 percentages, in combination with Feammox activity, may be additional indicators of defluorination by A6.

Aside from A6 and *rdhA* counts, PAA-coated goethite also had an impact on the microbial community, resulting in a distinct shift in the grouping of microbial communities, as shown in Figure S2. This is further exemplified at the phylum and family levels across the three treatments, as shown in Figures S3 and S4. Some notable shifts in microbial phyla include increases in the nitrogen-fixing bacteria Gaiellales and Xanthobacteraceae, an increase in Actinobacteria, and a decrease in Burkholderiaceae.^34,35^

### 3.2 ^12^C_8_–PFOS-Spiked Sediment Column Results

^12^C_8_–PFOS was spiked across all conditions to better achieve an F mass balance. Results show that amending PAA-coated goethite to the ^12^C_8_–PFOS-spiked columns had a positive effect on the Feammox process, A6 growth, and PFAS removal. Compared to the nonspiked columns, there was greater Feammox activity with NH_4_^+^ levels decreasing by 25.5 mg/L for Sed-2 C3 (Fe­(III) + NH_4_^+^ treatment) and by 9.7 mg/L for Sed-3 C3 (Fe­(III) + NH_4_^+^ treatment) over 50 days. A similar decrease in NH_4_^+^ was seen in the Sed-2 C2 (NH_4_^+^ treatment) but not in the Sed-3 C2 (NH_4_^+^ treatment) columns. The increase in pH was also comparable between the spiked and nonspiked columns, with Sed-2 C3 (Fe­(III) + NH_4_^+^ treatment) showing an increase in pH from 4.5 to 6, and Sed-3 C3 (Fe­(III) + NH_4_^+^ treatment) showing an increase in pH from 4.5 to 5.5^.–^

#### 3.2.1 PFOS Removal and F Mass Balance in the ^12^C_8_–PFOS-Spiked Sediment Columns

During the initial breakthrough period for NH_4_^+^ and PAA-coated goethite, 7.4 ± 0.3 mg (or 64%) and 5.4 ± 0.7 mg (47%) of PFOS were flushed from Sed-3 C2 and C3, respectively, and 1.0 ± 0.5 mg (17%) and 1.8 ± 0.3 mg (10%) of PFOS were flushed from Sed-2 C2 and C3, respectively. The higher amount of PFOS in the effluent of Sed-3 was due to its sandy properties and lower SOM content compared to the high clay and high SOM content of Sed-2. This difference in flushed PFOS in the effluent between Sed-2 and Sed-3 is consistent with the sorption results in Table S3, which show greater PFOS sorption to Sed-2 than to Sed-3. However, after 50 days of incubation, the mass of PFOS in the effluent decreased for all columns over the five flushed pore volumes. 0.66 ± 0.1 mg and 0.19 ± 0.16 mg of PFOS were flushed out from the Sed-3 C2 and C3 treatments, respectively, and 0.51 ± 0.3 mg and 0.16 ± 0.12 mg of PFOS were flushed from the Sed-2 C2 and C3 treatments, respectively.

Figure 4 shows the PFOS extracted from the soil Sed-2 and Sed-3 on day 0 and day 50 of the experiment, separated by treatment and column depth. On day 50, after flushing the columns for 5 pore volumes, the columns were disassembled and split into three sections (top, middle, and bottom), with each section undergoing PFOS soil extraction. These extractions showed a decrease in PFOS concentrations across all columns, with the greatest decrease seen in Sed-2 C3 (Fe­(III) + NH_4_^+^ treatment), followed by Sed-3 C3 (Fe­(III) + NH_4_^+^ treatment). Sorption tests, shown in Table S3, demonstrate that C3 with the amendment of PAA-coated goethite does not sorb more PFOS than C2 (NH_4_^+^ treatment). This may be because the PAA-coated goethite decreases the ζ-potential of the goethite, thus limiting its interaction with PFOS. Likewise, in these sorption tests, the total PFOS recovery in the system remained the same in both C2 and C3 for Sed-2 and Sed-3. PFOS mobilization and flushing from the column did occur, as shown in Table S4 for measurements conducted on day 0, during the initial breakthrough, and day 50, after pumping was resumed. On day 0, soil type played a predominant role, with Sed-3 flushing more PFOS than Sed-2; however, on day 50, the experimental condition played a larger role, with C2 (NH_4_^+^ treatment) flushing more PFOS than C3 (Fe­(III) + NH_4_^+^ treatment) (Table S4). On both days, PAA-coated goethite amendment resulted in less PFOS flushing. Overall, the PFOS recovery (measured as a sum of the PFOS in the effluent during the initial breakthrough and the flushing after pumping was resumed, plus the PFOS extracted from the sediments at the end of the experiment) was 77% and 23% for Sed-2 C2 and C3 treatments, respectively, and 91% and 50% for Sed-3 C2 and C3 treatments, respectively, as shown in Table S4. The much higher percentage of PFOS unaccounted for in the C3 (Fe­(III) + NH_4_^+^ treatment) vs the C2 (NH_4_^+^ treatment), despite similar sorption capacities, indicates the impact of PAA-coated goethite on enhancing the activity of A6 and possible PFOS biodegradation.

**Figure 4:**
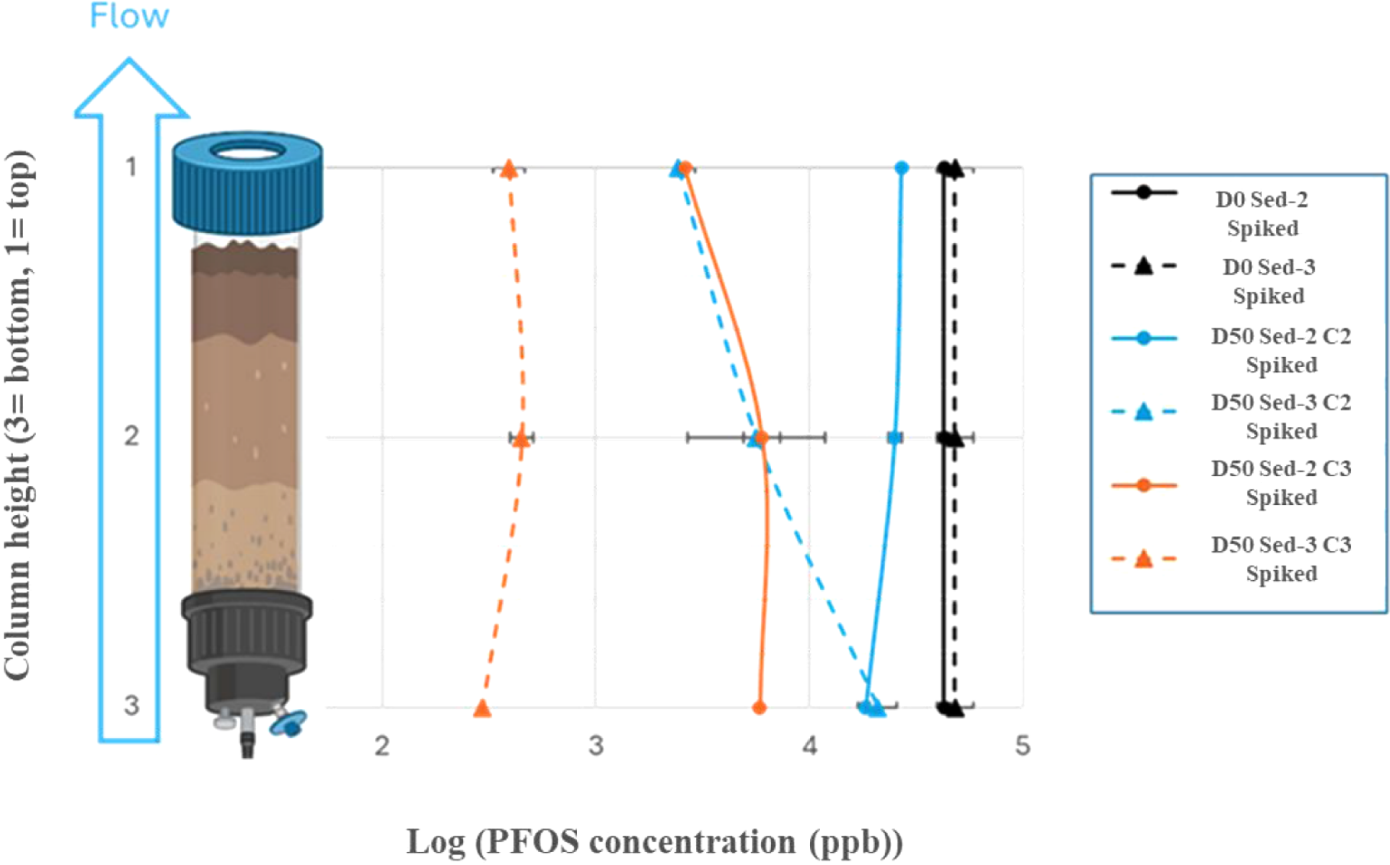
On day 50, after flushing for 5 pore volumes, the columns were disassembled and split into three sections. The results for the PFOS soil extractions are shown above. Here, the addition of NH_4_^+^ to the columns showed a decrease in extracted PFOS over 50 days in C2. This decrease in extractable PFOS was enhanced by the addition of Fe­(III) in C3.

Biodefluorination of PFOS is supported by the presence of F^–^ in the effluent of the C2 and C3 columns after 50 days of incubation. Compared to the nonspiked experiments, results indicate a 3-fold and 2-fold increase in F^–^ production for Sed-2 C2 and C3 treatments, respectively, and a 3-fold increase in F^–^ production for both Sed-3 C2 and C3 treatments (Table S5). Assuming that the only variable that was altered between the spiked vs nonspiked experiments was the addition of PFOS, the increase in F^–^ mass in the effluent can be attributed to the defluorination of the spiked PFOS. A final F mass balance of the additional PFOS for the columns is provided in Table S6 and shows a 93% and 42% F recovery for Sed-2 C2 and C3 treatments and a 97% and 59% F recovery for Sed-3 C2 and C3 treatments, respectively, thus showing that a percentage of the missing PFOS can be accounted for by the presence of F^–^. The lower F recovery in the C3 treatments with higher Fe­(III) levels is consistent with previous observations of decreased F recovery at higher pH and may also be affected by the possible presence of unknown intermediates.^30^

#### 3.2.2 ^12^C_8_–PFOS-Spiked Column A6 Numbers

The ^12^C_8_–PFOS-spiked columns showed similar A6 counts for C2 (NH_4_^+^ treatment) but higher A6 counts for C3 (Fe­(III) + NH_4_^+^ treatment) compared to the nonspiked columns, as shown in Figure S5. For Sed-2 C3, there was a 3.5× increase in A6 numbers compared to that of the pretreatment sediments and a 5-fold increase in A6 numbers compared to the nonspiked columns. Conversely, there was only a slight increase in A6 numbers for the Sed-3 C3 (Fe­(III) + NH_4_^+^ treatment) spiked columns compared to nonspiked columns and a decrease in A6 numbers compared to the pretreatment sediments, which once again suggests a role of sediment type in promoting the growth of A6. However, as stated previously, A6 numbers may not be the best measure of A6 activity. Therefore, *rdhA* gene counts were measured and showed a 2× increase in Sed-2 C3 and a 1.1× increase in Sed-3 C3 over the 50-day incubation. The increase in both A6 counts and *rdhA* gene counts for both Sed-2 C3 and Sed-3 C3, combined with the decrease in total extractable PFOS and increased F^–^ production, suggests that PAA-coated goethite was instrumental in enhancing A6 activity and PFOS defluorination.

### 3.3 ^13^C_8_–PFOS-Spiked Column Results

#### 3.3.1 ^13^C_8_–PFOS A6 Activity

^13^C_8_–PFOS was used to help determine the production of PFOS intermediates not attributable to precursors or preexisting shorter-chained PFAS. Because of the high cost of ^13^C_8_–PFOS and because a F mass balance experiment was already performed using ^12^C_8_–PFOS, a lower concentration of ^13^C_8_–PFOS was used for this experiment. As in the previous two experiments, Feammox activity was enhanced when the columns were treated with PAA-coated goethite, as shown by decreased NH_4_^+^ concentrations and increased pH. For this experiment, the overall Feammox activity was similar to that of the nonspiked column experiment for Sed-2, with approximately 15 mg/L NH_4_^+^ removal for Sed-2 C3 (Fe­(III) + NH_4_^+^ treatment) and 3 mg/L for Sed-3 C3 (Fe­(III) + NH_4_^+^ treatment). Likewise, the pH for Sed-2 increased from 4.5 to 6 for C3 (Fe­(III) + NH_4_^+^ treatment) and from 4.5 to 5 for C2 (NH_4_^+^ treatment); however, for Sed-3, the pH only increased to 5 for both treatments. F^–^ was measured, and the concentration increased compared to the nonspiked controls; however, given the much lower PFOS concentration compared to the ^12^C_8_–PFOS-spiked experiment discussed above, it was only used qualitatively as an indicator of defluorination across all treatments (Table S5).

As shown in Figure S5, PAA-coated goethite treatment increased the A6 counts/g sediment in the ^13^C_8_–PFOS-spiked sediment for both Sed-2 and Sed-3. The A6 count for Sed-2 C3 (Fe­(III) + NH_4_^+^ treatment) was similar to that of the PFOS-spiked sediment experiment but higher than the nonspiked sediment experiment. The opposite was true for Sed-3 C3 (Fe­(III) + NH_4_^+^ treatment), where the nonspiked sediments had one magnitude greater A6 counts than the PFOS-spiked columns and nearly two magnitudes greater A6 counts than the ^13^C_8_–PFOS-spiked columns. This shows that A6 numbers may be highly dependent on the iron availability, PFAS concentrations, and sediment type. A6 defluorination activity, as shown by the *rdhA* gene counts, showed a 2-fold increase and a 1.3 times increase for Sed-2 and Sed-3, respectively.

#### 3.3.2 ^13^C_8_–PFOS Intermediates

Effluent results show a significant amount of ^13^C_8_–PFOS was lost through flushing, especially during the breakthrough period before the 50-day incubation. During that time, there were no ^13^C_8_–PFOS intermediates present in the effluent. After 50 days of incubation, the presence of ^13^C_8_–PFOS in the effluent decreased, and the presence of degradation intermediates was detected in the Sed-2 C2 (NH_4_^+^ treatment) and C3 (Fe­(III) + NH_4_^+^ treatment) columns, as well as in the Sed-3 C3 (Fe­(III) + NH_4_^+^ treatment) columns. By scanning the intermediate peaks at various mass-to-charge (*m*/*z*) ratios and verifying these values using standards, peaks were detected for *m*/*z* values of 455.99 ([M–H]–1) and 213.99 ([M–H]–1), which corresponded to ^13^C_7_–PFHpS and ^13^C_4_–PFBA, respectively (Figure S6). No corresponding signals were observed for day 0 samples or in any of the control columns.

^13^C_7_–PFHpS was detected in both Sed-2 C2 (NH_4_^+^ treatment) and Sed-2 C3 (Fe­(III) + NH_4_^+^ treatment) columns at concentrations of 4.2 ± 1.2 μg/L and 9.6 ± 2.3 μg/L respectively. Interestingly, ^13^C_4_–PFBA was only detected in Sed-3 C3 (Fe­(III) + NH_4_^+^ treatment) at a concentration of 1 μg/L. Previous studies (Jaffé et al., 2021) have shown the production of shorter-chain PFAAs (C7–C4) during the degradation of PFOA/PFOS, although detection of the individual PFAAs varies between experiments and sampling points, as is also observed in these column experiments, indicating that the buildup of some of these intermediates is transient, since they have also been shown to be degradable by A6.^19^

Whereas ^13^C intermediates were detected in the effluents, none were detected in the sediment extracts, which was attributed to their lower sorption coefficient (K_d_) relative to PFOS. The lowest concentration of ^13^C_8_–PFOS in the sediment extract was found in Sed-3 C3 (Fe­(III) + NH_4_^+^ treatment) at 281 ± 20 μg/kg. Both Sed-2 conditions had similar ^13^C_8_–PFOS concentrations of 329 ± 15 μg/kg and 340 ± 16 μg/kg for C2 (NH_4_^+^ treatment) and C3 (Fe­(III) + NH_4_^+^ treatment), respectively.

## 4 Discussion

### 4.1 A6 Activity in AFFF-Impacted Sediment Columns Stimulated with PAA-Coated Goethite

While previous experiments with Sed-2 and Sed-3 by Huang et al. (2024b) demonstrated that, in smaller laboratory-scale slurry incubations, Fe­(III) addition resulted in greater A6 activity in sediments with lower background Fe­(III) levels, the present study reveals that Fe­(III) retention and transport in the sediment, as governed by sediment organic matter content and composition, are also critical determinants of A6 activity.^20^ The clay sediments of Sed-2, for example, had a greater Fe­(III) retention than the sandy sediments of Sed-3. As a result, the increased Fe­(III) retention in the column appears to have resulted in greater A6 activity in Sed-2 compared to the sandy sediment of Sed-3, despite having a higher initial Fe­(III) content in Sed-2. The increase in A6 activity resulted in higher PFAS defluorination in the PFOS-spiked columns, as shown by a 12% and 8% increase in F^–^ production in C3 treatments for Sed-2 and Sed-3, respectively, compared to the nonspiked sediments and the non-PAA-coated goethite columns.

When compared to the results of Huang et al. (2024b), in which the same sediments, Sed-2 and Sed-3, were tested in laboratory batch incubations under similar experimental conditions (DIW, NH_4_^+^ treatment, and Fe­(III) + NH_4_^+^ treatment), the laboratory batch incubations had significantly more PFAS defluorination as measured by F^–^ production.^20^ This may be due to the different Fe­(III) to sediment ratios in the two experimental setups. In Huang et al. (2024b), the ratio of Fe­(III) to sediment was 0.06 mmol of ferrihydrite/g of sediment. In comparison, the maximum Fe­(III) to sediment ratio that could be retained after breakthrough was 0.0008 mmol of goethite/g for Sed-2 and 0.0005 mmol of goethite/g for Sed-3. This lower Fe­(III)-to-sediment ratio likely accounts for the reduced Feammox activity and PFAS defluorination observed in the column experiments relative to the laboratory batch incubations, as well as the differences between Sed-2 and Sed-3, and indicates that strategies for delivering higher Fe­(III) loadings to subsurface environments merit further investigation.

### 4.2 ^13^C_8_–PFOS-Spiked Intermediates

Considerable work remains to elucidate the mechanism of PFOS defluorination by A6. This paper sheds light on potential degradation products by tracking detectable ^13^C_8_–PFOS intermediates. These experiments showed the presence of ^13^C_7_–PFHpS and ^13^C_4_–PFBA, accompanied by an increase in F^–^ concentrations in the effluent after 50 days of sediment column incubations. These compounds, previously shown to be produced by A6 ^19^ during the degradation of PFOS, were determined using their respective *m*/*z* ratios and retention times and, in addition to the production of F^–^, provide evidence that PFOS is being degraded in the biostimulated column experiments.

Further analyses employing PDA, MS/MS, or NMR spectroscopy, which were beyond the scope of the present study, could provide additional mechanistic insights into unidentified intermediates and novel transformation products that were not detected herein.

### 4.3 Implications for Pilot Field Studies

The results of this soil column biostimulation study suggest that scaling up the results from batch slurry A6 biostimulation experiments for PFAS degradation to a small field test is warranted in soils containing A6. The soils selected for this study were acidic sediments screened for A6, and it is unlikely that we could have achieved these biostimulation results under alkaline conditions. Therefore, initial soil screening and maintenance of an acidic pH during the remediation process are important.

Further studies are needed to determine the delivery method of PAA-coated goethite to the soil, the appropriate iron dosage regimes, the optimal soil moisture concentration, and the subsurface redox conditions under in situ conditions.

## Supplementary Material



## Notes

The sediments, the relationship between PAA-coated goethite concentration and absorbance, bacterial nutrient composition, description of chemical and microbial analyses, results of microbial community analysis, PFOS sorption experiments/results, F mass balances, and sample chromatograms from the LC–MS analyses (DOCX)
