# Supplementary material for: Stimulation of Acidimicrobium sp. Strain A6 Biodegradation of PFOS in AFFF-Impacted Sediment Columns Using PAA-Coated Goethite
Source: ACS ES T Eng. 2026 Jun 30;6(7):1994–2003. doi: 10.1021/acsestengg.6c00272 (PMC13366567; doi:10.1021/acsestengg.6c00272)
Supplement: Supplementary file 1 [file ee6c00272_si_001.docx]

Supplemental Information

**Stimulation of Acidimicrobium sp. Strain A6 Biodegradation of PFOS in AFFF Impacted Sediment Columns using PAA-Coated Goethite**

**Matthew W. Sima, Shan Huang, Peter R. Jaffé**

Table S1. NH_4_^+^, Fe(II/III), pH, TOC, and sediment composition data for Sed-2 and Sed-3

|  | NH_4_^+^ (mg/kg) | Fe(II) (mg/kg) | Fe(III) (mg/kg) | pH | TOC (mg/kg) | Soil Composition |
| --- | --- | --- | --- | --- | --- | --- |
| Sed-2 PFAS Impacted soil | 5.08 | 67.02 | 61.19 | 6.04 | 117.8 | Clay |
| Sed-3 PFAS Impacted soil | 5.34 | 23.1 | 20.3 | 6.57 | 23.5 | Silt |

Table S2. PFAS concentrations measured and reported by SGS Canada, for Sed-2 and Sed-3 in ug/Kg

| **Sample ID** | **4:2 FTS** | **6:2 FTS** | **8:2 FTS** | **N-EtFOSAA** | **N-MeFOSAA** | **PFBA** | **PFPeA** | **PFHxA** | **PFHpA** | **PFOA** | **PFOSA** | **PFNA** |
| --- | --- | --- | --- | --- | --- | --- | --- | --- | --- | --- | --- | --- |
| Sed-3 | 0.69 | 55.02 | 21.65 | 0.98 | 1.95 | 0.58 B | 3.24 | 11.32 | 4.51 X | 35.23 X, B | 13.78 X | 2.1 X |
| Sed-2 | 1.14 | 195.69 X | 227.97 | 1.36 | 0.56 | 1.86 B | 8.24 B | 12.75 B | 7.97 | 23.08 | 756.54 B, E | 33.03 B |

| **Sample ID** | **PFDA** | **PFUdA** | **PFDoA** | **PFTrDA** | **PFTeDA** | **PFBS** | **PFPeS** | **PFHxS** | **PFHpS** | **PFOS** | **PFNS** | **PFDS** |
| --- | --- | --- | --- | --- | --- | --- | --- | --- | --- | --- | --- | --- |
| Sed-3 | 0.38 | 0.32 | 0.27 | 0.1 X | 0.11 X | 25.32 | 53.41 | 1012.08 E | 2388.35 X,E | 2590.17 X, E, B | 88.25 X | 8.78 X |
| Sed-2 | 6.17 | 1.37 B, X | 1.17 X | 0.06 X | 0.13 X | 5.2 | 6.2 | 199.74 | 119.77 X | 1376.77 X, E | 15.17 X | 14.37 X |

X: Corresponding internal standard recovery was outside of the acceptable range (50-150%).

J: The reported result is an estimate. The value is less than the minimum calibration level but greater than the estimated detection limit (EDL).

B: This flag is used when the analyte is found in the associated blank, as well as in the sample. Background concentrations were subtracted from the impacted matrices.

E: Concentrations exceeding the calibration range.

**Calibration for PAA-Coated Goethite Quantification in the Column Effluent:**


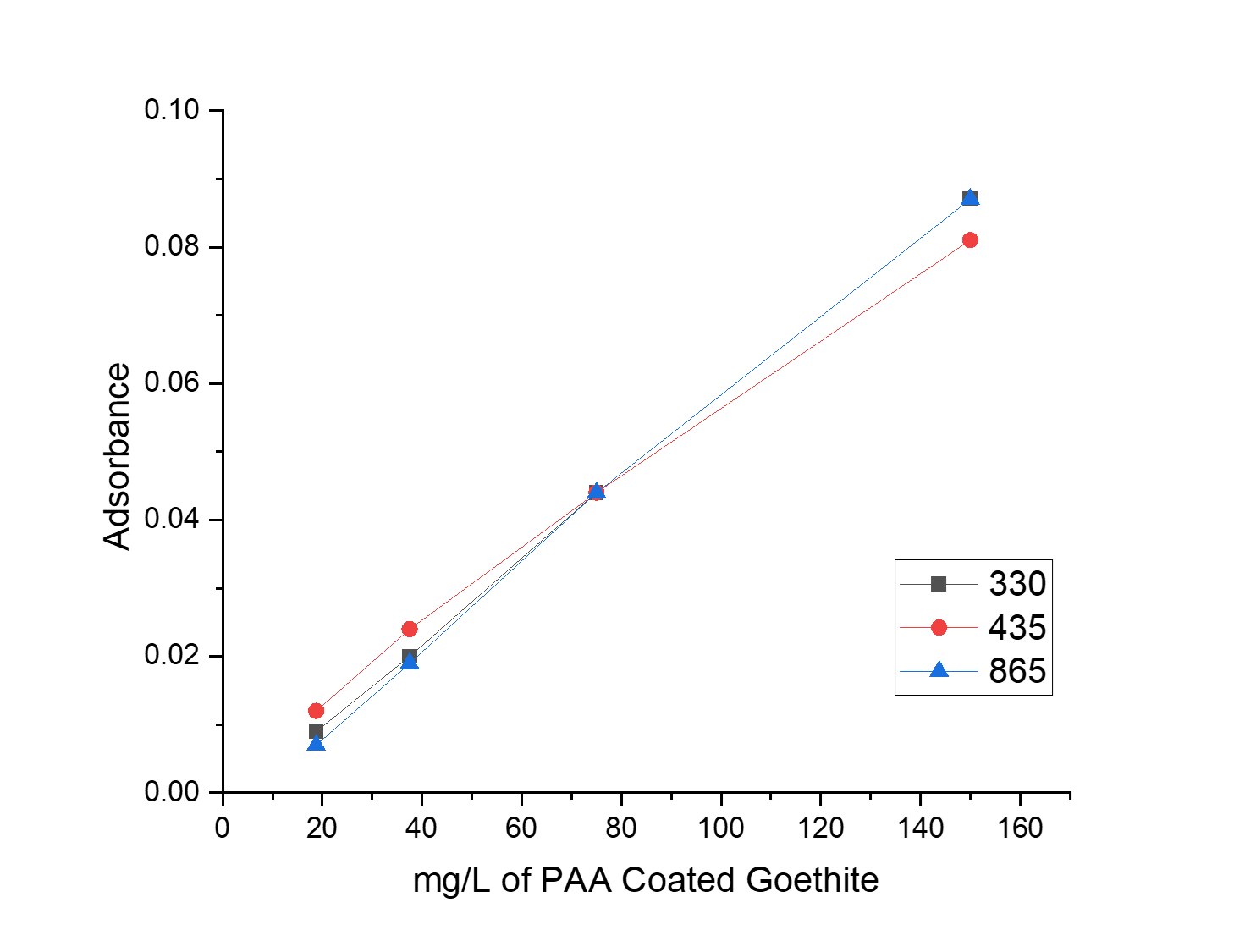


Fig. S1. The absorbance of different standard concentrations of PAA-coated goethite at different wavelengths was linear within the expected concentration range

**Bacterial nutrient medium composition:**

The bacterial nutrient medium was adopted from Huang et al., 2024b and consisted of 128.4 mg/l NH_4_Cl, 79.2 mg/l (NH_4_)_2_SO_4_, 16.8 mg/l NaHCO_3_, 70.1 mg/l KHCO_3_, 13.6 mg/l KH_2_PO_4_, 11.1 mg/l CaCl_2_, 1 ml/l of trace element mixture (consisting of nitriloacetic acid, 4.5 g/l; FeCl_2_ · 4H_2_O, 0.4 g/l; MnCl_2_ · 4H_2_O, 0.1 g/l; CoCl_2_ · 6H_2_O, 0.17 g/l; ZnCl_2_, 0.1 g; CaCl_2_, 0.2 g/l; H_3_BO_3_, 0.019 g/l; and sodium molybdate, 0.01 g/l and adjusted to a pH of 7 with KOH), and 1 ml/l of vitamin mixture (consisting of biotin, 0.002 g/l; folic acid, 0.002 g/l; pyridoxine hydrochloride, 0.01 g/l; thiamine hydrochloride, 0.005 g/l; riboflavin, 0.005 g/l; nicotinic acid, 0.005 g/l; pantothenic acid 0.005 g/l; B_12_, 0.0001 g/l; *p*-aminobenzoic acid, 0.005 g/l; thioctic acid, 0.005 g/l).

**Chemical analyses**

Anion and cation analyses were conducted using a Dionex™ Ion Chromatograph (IC3000) with an AS-18 and CS-16 column, respectively. To confirm the F^-^ results, select samples were tested using a perfectION™ combination electrode (Mettler-Toledo, USA).

In-house PFAS analyses were conducted using an LCMS-2050 Single Quadrupole Mass Spectrometer (Shimadzu), in accordance with EPA Method 537.1 (1, 2). The column used during analysis was a Shimpak Velox C18 column (I.D. 2.1 mm, length 50 mm, particle size 2.7 μm) from Shimadzu at an eluent flow rate of 0.4 mL/min at 40^o^C thus enabling a better separation between the long and short chained PFAS. The eluent was methanol/DI spiked with ammonium acetate. The gradient for the mobile phase in liquid chromatography was programmed as follows: initially, the mobile phase consisted of 95% solvent A (5 mM ammonium acetate in water) and 5% solvent B (HPLC-grade methanol), maintained for 0.5 mins. The concentration of solvent B was then gradually increased to 95% over 12 mins and held steady for 2 mins, was subsequently reduced to 10% in 1 min, and was finally maintained for an additional 4 mins. The mass spectrometer operated in negative-ion electrospray mode. Quantitative analysis relied on certified standards for native compounds and isotope-labeled internal standards, obtained from Wellington Laboratories, Inc., with a purity of 98% or higher. A more detailed explanation of the methods used is available in Huang et al. (3).

PFAS soil extraction and analyses were conducted as described by Chiavola et al. (4) and Huang et al (3). At end of each experiment, the column was split into thirds and PFAS from sediment samples in each third were extracted with methanol in a one-to-one dilution with 5g of sediment sample mixed with 5ml of 50% diluted methanol solution. The slurry mixture was then sonicated in a water bath for 15 min at 60 °C to extract the sorbed PFAS from the sediment samples and then centrifuged for 10 minutes at 15,000x *g* to separate out the solids*.* The supernatant was then filtered through 0.22 μm membrane filter into glass 1.5 ml LC vials for PFAS analysis. This method extracts both the dissolved and the sorbed PFAS in the liquid and solid phases and has been applied previously in Huang et al. (3) and Chiavola et al. (4) with acceptable PFAS recovery rates of 70-130%.

In addition to the in-house analysis, selected sediment samples from the non-spiked sediment experiment were sent to SGS Canada for comparison and to SGS Shanghai for microbial community analyses. For these samples, 5 g of sediment from each third of the column (top, middle, and bottom) were mixed thoroughly before sending to SGS Canada for extraction using EPA Method 1633. After extraction, the samples were analyzed using the SGS method MLA-110 Rev 02.13. In general, our in-house PFAS measurements agreed with those from SGS Canada in the range of ±8% with only a few samples showing higher PFAS concentrations than those reported by SGS Canada.

**Microbial analyses**

DNA was extracted from the samples using the Fast DNA™ spin kit for soil (MP Biomedicals) and the concentration and quality checked using a Qubit® 2.0 Fluorometer (Thermo Scientific). qPCR was conducted in triplicate using the One Step SYBR PrimeScript RT-PCR Kit II (TaKaRa, Japan) following the manufacturer's instructions on a 96-well StepOnePlus Real-Time PCR System (Applied Biosystems, CA, USA). The sequence used to identify the A6 bacteria was the V4 region of the 16S rRNA gene of bacteria was amplified using primer-set 515f-806r (5). The PCR amplification efficiencies were 92–112 %, and the correlation coefficients (R^2^) for all assays were > 0.99.Metagenomic data were analyzed by SGS Shanghai using Method SPSS v12 on an Illumina MiSeq platform at Novogene Co. Various diversity and richness indices were estimated including Observed-species, Chao1, Shannon, Simpson, ACE, Goods-coverage according to QIIME2 version 2022.11. Microbial communities were further analyzed using Principal coordinate analysis (PCoA) and permutational multivariate analysis of variance (Adonis) to show the similarity of the microbial community across columns and locations (section adopted from 3).

**Oxygen Consumption Test:**

Oxygen consumption over 5 days was measured following the standard Hach Method 8043 (Hach Company, Loveland, CO, USA) (6). Ten grams of wet sediment were mixed with dilution water to obtain a 300 mL slurry in a BOD bottle. The initial dissolved oxygen (DO₁) was determined with a Hach LDO® probe (Method 10360). Samples were then incubated in the dark at 20 ± 1 °C for five days, after which the final DO (DO₅) was measured. BOD₅ values were

The initial dissolved oxygen (DO) concentration was 4.2 mg/L and decreased to 0.2 mg/L after five days of incubation. The oxygen consumption of the soil was approximately 0.12 mg O₂ g⁻¹, indicating a moderate to high level of organic matter. This SOM content was sufficient to support microbial respiration and lead to oxygen depletion over time.

**Further Analyses of the Effect of the Stimulations on the Microbial Communities:**

The effect of PAA-coated goethite on the bacterial community is presented in Fig. S2 where the principal coordinate analysis shows that 53.7% of the difference in community can be attributed to the amendment of PAA-coated goethite. PAA-coated goethite amended C3 columns show a clear shift in microbial community compared to the DIW control C1 and the NH_4_^+^ control C2. The Sed-3 column shows a greater gradient with the C1, C2, and C3 communities visibly distinct from each other, compared to the Sed-2 columns, where there was little difference between C1 DIW and C2 NH_4_^+^ treatment communities. In fact, the C3 Fe(III) columns showed greater similarity to the day 0 columns compared to the other treatment conditions, despite undergoing flushing.


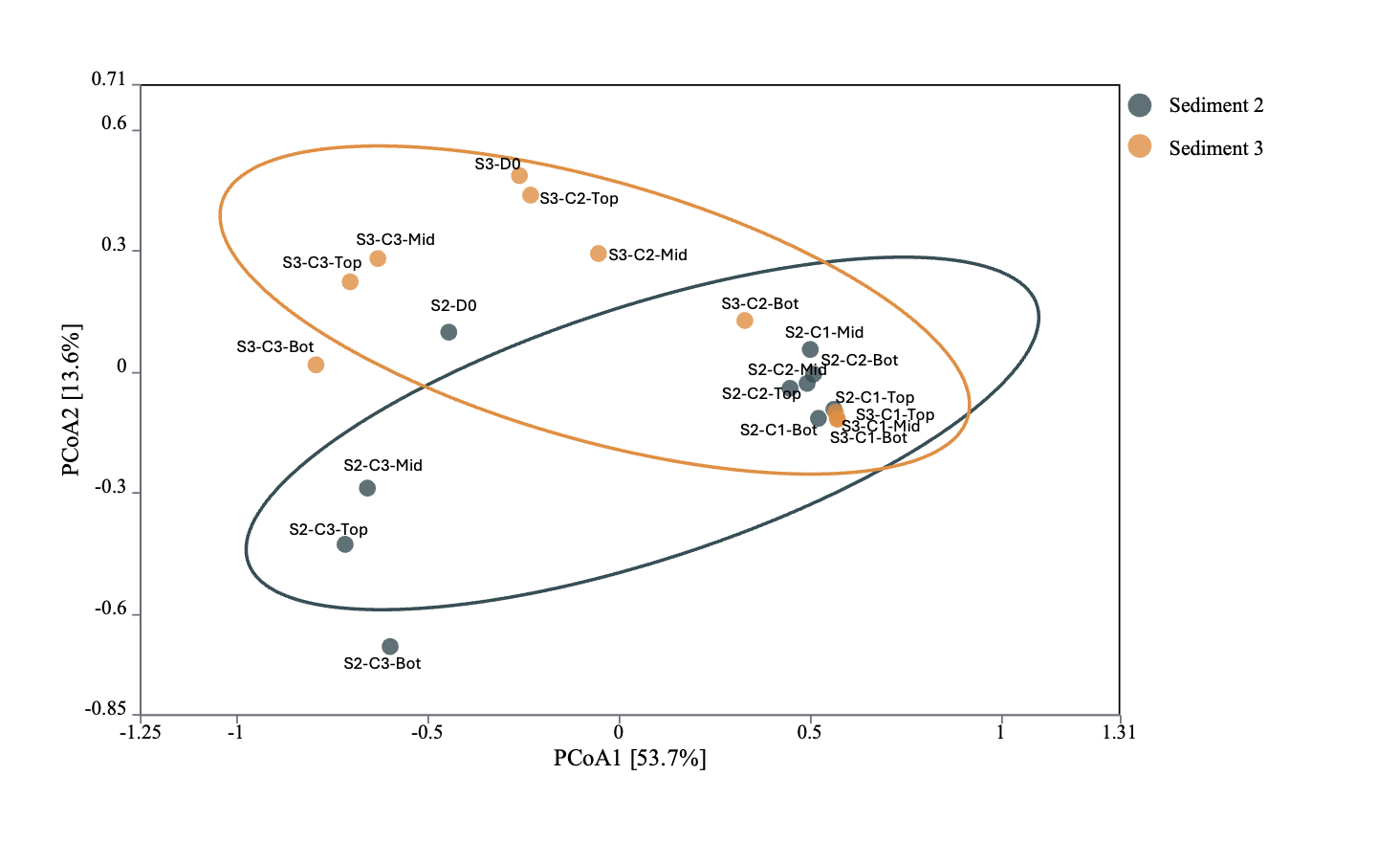


Fig. S2. Principal component analysis showing divergence of bacterial communities across sediment type, iron amendment, and column location (C1 is the DIW control; C2 is the NH_4_^+^ no-iron treatment; C3 is the Fe(III) and NH_4_^+^ experimental treatment)

The beneficial effect of PAA-coated goethite on microbial community diversity is further supported by the results presented in Fig. S3 which shows a relative abundance graph for the top bacterial phyla and Fig. S4 which shows the phyla tree for the top 20 phyla. The day 0 microbial communities for both Sed-2 and Sed-3 were comparable to those seen in high PFAS sediments with Proteobacteria, Acidobacteria, Actinobacteria, Chloroflexi, and Bacteridota among the top 10 phyla as shown in Fig. S3 (7). After 50 days, the relative abundance of Actinobacteria increased across all conditions while the proteobacteria decreased with the addition of PAA-coated goethite. The addition of PAA-coated goethite further resulted in a more even distribution between the top 10 bacterial phyla while C1 (DIW) and C2 (NH_4_^+^) treatments were primarily dominated by proteobacteria Fig. S3.


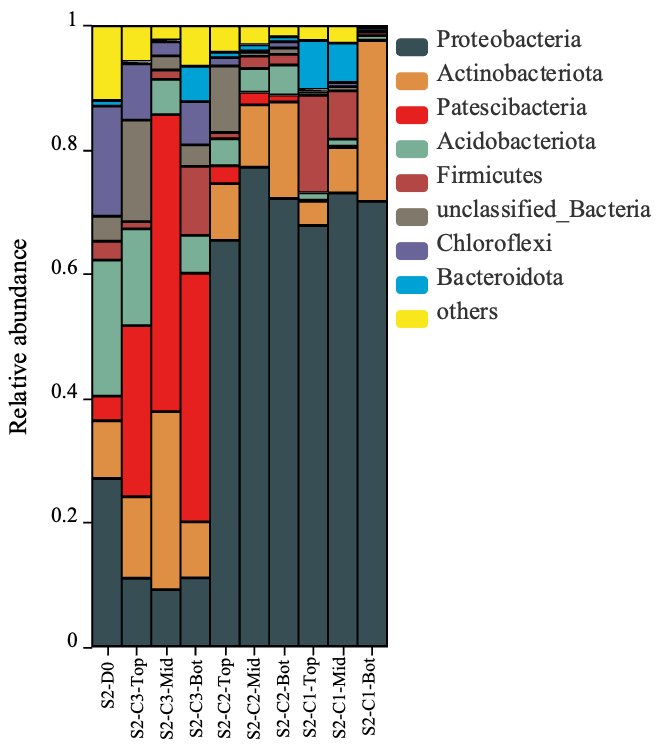

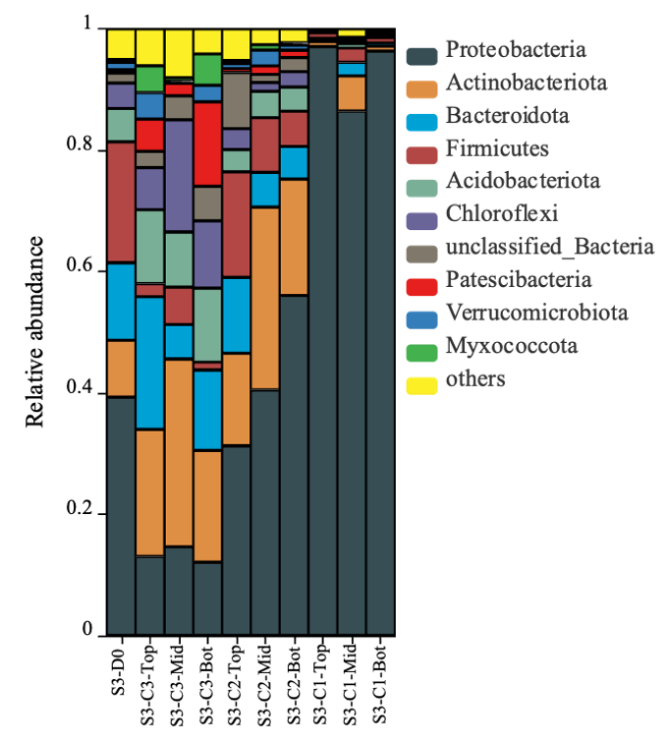


Fig. S3. Relative abundance of the top phyla for each column section (C1 is the DIW control; C2 is the NH_4_^+^ no-iron treatment; C3 is the Fe(III) and NH_4_^+^ experimental treatment)


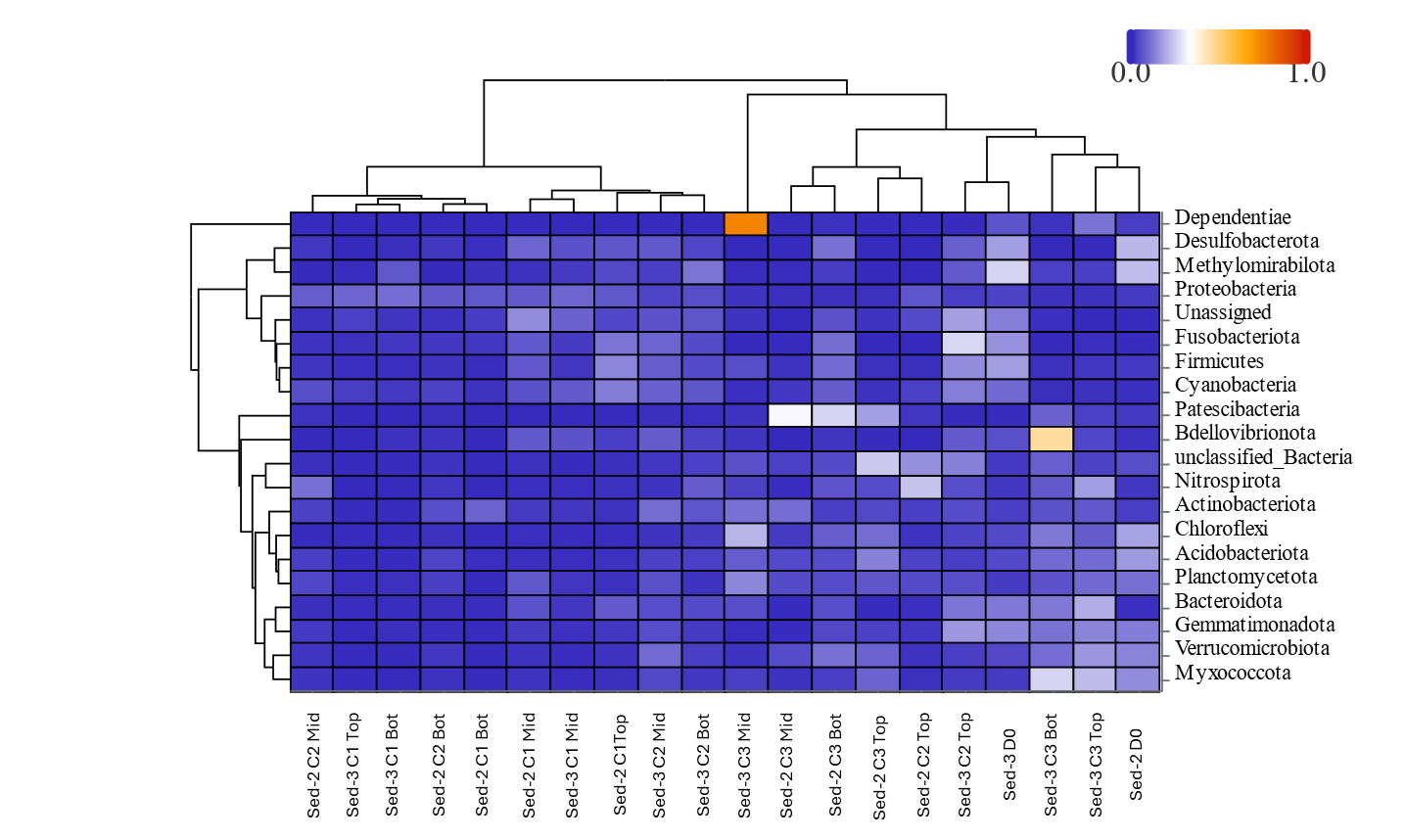


Fig. S4. Microbial Community breakdown by phylum showing a clear difference in microbial communities between C3 vs C2 and C1. (C1 is the DIW control; C2 is the NH_4_^+^ no-iron treatment; C3 is the Fe(III) and NH_4_^+^ experimental treatment)

When analyzed by families, the microbial communities show a distinct shift between the day 0 sediments and the day 50 sediments for each condition and sediment type. For day 0, the microbial data for both sediments are representative of common AFFF impacted soils with the presence of Acidobacteriaceae, Burkholderiaceae, JG30-KF-CM45, Rhizobiaceae, and Chloroflexi being some of the more dominant families (7, 8, 9, 10, 11). Most notably previous studies have shown a relationship between select Burkholderiaceae and the ability to degrade anthropogenic halogenated pollutants such as pentachlorophenol, although their ability to defluorinate PFAS needs further investigation (12).

After 50 days of incubation under the different conditions C1 (DIW), C2 (NH_4_^+^), and C3 (Fe(III) + NH_4_^+^ treatment), the bacterial communities show a distinct shift between the three conditions. The C1 samples display significantly lower bacterial numbers across all families, except for Burkholderiaceae, which show an order of magnitude increase from day 0 in both Sed-2 and Sed-3. A similar trend was observed for Burkholderiaceae in the C2 columns, which show a slightly lower but still statistically significant increase. This was accompanied by an increase in nutrient cycling and nitrogen fixating bacteria such as Xanthobacteraceae and Gaiellales (7, 13). For both the C1 and C2 columns, Burkholderiaceae was the dominant family and displayed the greatest bacterial community increase regardless of column section (top, middle or bottom).

For the C3 columns, the addition of PAA-coated goethite led to a more uniform and diverse spread of bacterial numbers across the families. Most notably, however, there was a significant drop of over two magnitudes in Burkholderiaceae numbers over the incubation period in both Sed-2 and Sed-3. There was also an increase in the family Saccharimonadales, which are known for their role in phosphorus cycling in wastewater treatment (14). These shifts in bacterial communities further show that while the amendment of PAA-coated goethite may be beneficial for bacterial diversity across most families, its amendment did adversely affect Burkholderiaceae. If and how this decrease in Burkholderiaceae might affect the transformation of polyfluorinated PFAS at AFFF sites augmented with Fe(III) needs further investigation.

Changes in the microbial diversity in response to the stimulation was further determined using the Chao and Shannon diversity indices. Both sediment types showed a decrease in microbial diversity from the day 0 sediments regardless of treatment, however, a greater Chao diversity index was seen in the C3 (Fe(III)) columns with Chao values of 1373 and 1583.2 for Sed-2 and Sed-3, respectively, compared to the C1 (DIW) and C2 (NH_4_^+^) treatments with Chao values of 557.6 and 718.3 for Sed-2 and 404.4 and 873.7 for Sed-3, respectively. This is also mirrored in the Shannon index except for the Sed-2 C3 (Fe(III)) column which showed a high Chao index but low Shannon index, which suggests that although there may be many species, there is an uneven distribution of species abundance across the community especially in the bottom and middle sections of the columns.

- 1. PFOS Sorption Test

To test both the sorption and the extractability of PFOS from the Sed-2 and Sed-3 sediments, experiments were conducted using 1ppm of PFOS in 5 grams of Sed-2 and Sed-3 sediments, respectively, over 48 hours under the three conditions C1 (DIW), C2 (NH_4_^+^ amended), and C3 (Fe(III)+NH_4_^+^ amended). The results showed that SOM was important for both sorption and extractability of PFOS, with greater SOM resulting in greater sorption and lower extractable PFOS. Here, greater PFOS sorption was seen in Sed-2 than Sed-3 as shown by a lower concentration of PFOS in the dissolved phase. Across conditions, there was the greatest PFOS sorption in C2 and C3 and the least sorption in C1 (DIW) (Table S3).

Table S3. Sorption experiment for Sed-2 and Sed-3 using medium from conditions C1, C2, and C3 after adding 1ppm of PFOS.

|  | Sediment 2 | | | Sediment 3 | | |
| --- | --- | --- | --- | --- | --- | --- |
|  | C1 (DIW) | C2 (NH_4_^+^ amended) | C3 (Fe(III)+NH_4_^+^ amended) | C1 (DIW) | C2 (NH_4_^+^ amended) | C3 (Fe(III)+NH_4_^+^ amended) |
| Dissolved PFOS (ug/L) | 425.17 ±  41.84 | 270.65 ±  40.4 | 262.43 ±  13.34 | 674.68 ±  19.39 | 286.95 ±  12.44 | 412.13 ± 36.09 |
| Extracted PFOS (ug/L) | 444.58 ±  25.04 | 432.91 ±  47.97 | 391.62 ±  34.21 | 414.35 ±  19.10 | 508.19 ±  4.29 | 437.05 ± 25.56 |
| Total PFOS (ug/L) | 869.75 ±  48.7 | 703.56± 62.7 | 654.05±  36.7 | 1,089.03 ±  27.2 | 795.14 ± 13.2 | 849.19 ±  44.2 |

The extractable PFAS showed that PFOS sorption to the sediment increased with the addition of NH_4_^+^ but was not affected by the presence of PAA-coated goethite. This can be because the negatively charged PFOS was attracted to the positively charged NH_4_^+^ while the lower zeta potential of the PAA-coated goethite repelled the PFOS. However, even without the PAA-coating, previous sorption experiments with Fe(III) in the presence of the Feammox medium showed little sorption of PFOA/PFOS.

**^12^C_8_-PFOS Spiked Sediment Column PFAS and F Mass Balance**

Table S4. PFOS mass balance in ^12^C_8_-PFOS spiked columns showing a higher percentage of PFOS unaccounted for in the C3 (Fe(III)) vs the C2 (NH_4_^+^) treatments.

| Condition | Initial PFOS (mg) | Total PFOS in outflow D0 (mg)* | Total PFOS in outflow D50 (mg)** | PFOS in column (mg) | Total PFOS Recovered (mg) | Approximate  % PFOS recovered |
| --- | --- | --- | --- | --- | --- | --- |
| Sed-2 C3 total PFOS | 10.3 ± 1.6 | 1.04 ± 0.3 | 0.16 ± 0.1 | 1.21 ± 0.2 | 2.41 ± 0.4 | 23.4 |
| Sed-3 C3 total PFOS | 11.5 ± 2.4 | 5.42 ± 0.2 | 0.19 ± 0.1 | 0.1 ± 0.05 | 5.70 ± 0.2 | 49.6 |
| Sed-2 C2  total PFOS | 10.3 ± 1.6 | 1.79 ± 0.3 | 0.51 ± 0.2 | 5.65 ± 0.3 | 7.95 ± 0.4 | 77.1 |
| Sed-3 C2  total PFOS | 11.5 ± 2.4 | 7.36 ± 0.5 | 0.67 ± 0.2 | 2.41 ± 0.5 | 10.4 ± 0.7 | 90.8 |

*This is the sum of PFOS in all of the collected samples over the 10 flushed pore volumes prior to the start of the experiment (D0)

**This is the sum of PFOS in all of the collected samples over the 5 flushed pore volumes after the 50-day incubation period (D50)

Table S5. F^-^ production in column experiments shows greater F^-^ production in the PFOS-spiked experiments compared to the non-spiked experiments.

| Condition | F^-^ in effluent from non-spiked columns (mg) | F^-^ in effluent from ^12^C_8_-PFOS spiked columns (mg) | F^-^ in effluent from ^13^C_8_-PFOS spiked columns (mg) |
| --- | --- | --- | --- |
| Sed-2 C3 total PFOS | 0.40 ± 0.03 | 1.64 ± 0.5 | 0.50 ± 0.05 |
| Sed-3 C3 total PFOS | 0.23 ± 0.02 | 0.95 ± 0.3 | 0.30 ± 0.04 |
| Sed-2 C2  total PFOS | 0.54 ± 0.03 | 1.61 ± 0.4 | 0.58 ± 0.07 |
| Sed-3 C2  total PFOS | 0.14 ± 0.01 | 0.61 ± 0.2 | 0.18 ± 0.04 |

Table S6. F mass balance in ^12^C_8_-PFOS spiked columns shows greater F^-^ produced for Sed-2 and greater F recovery in the C2 columns.

| Condition | total F added to the system (mg) | F in additional PFOS recovered (spiked – unspiked) (mg) | F^-^ in effluent corrected for non-spiked (mg) | Total F recovered | % F recovery |
| --- | --- | --- | --- | --- | --- |
| Sed-2 C3 | 6.65 ± 1.0 | 1.56 ± 0.4 | 1.24 ± 0.5 | 2.80 ± 0.6 | 42.0 |
| Sed-3 C3 | 7.43 ±1.6 | 3.68 ± 0.2 | 0.72 ± 0.3 | 4.40 ± 0.4 | 59.2 |
| Sed-2 C2 | 6.65 ± 1.0 | 5.13 ± 0.5 | 1.07 ± 0.4 | 6.20 ± 0.6 | 93.2 |
| Sed-3 C2 | 7.43 ±1.6 | 6.74 ± 0.8 | 0.47 ± 0.2 | 7.21 ± 0.8 | 97.1 |

**Spiked Sediment Column A6 numbers**


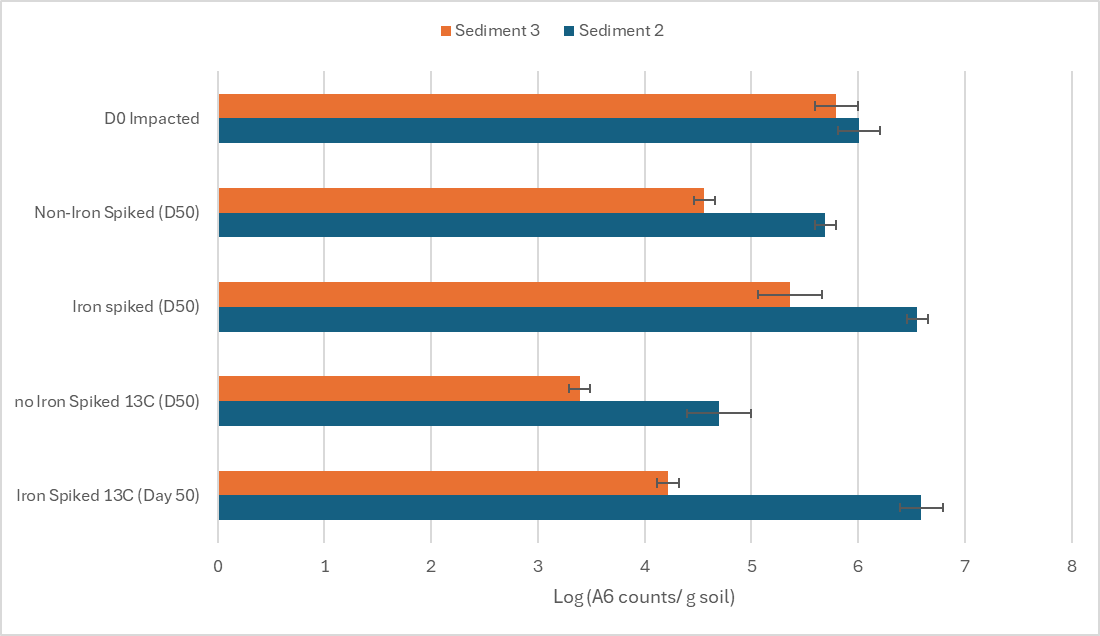


Fig. S5.Log (A6 counts/g sediment) for ^13^C_8_-PFOS and ^12^C_8_-PFOS Spiked Sediment Experiments

**^13^C Intermediate LC-MS Peaks:**


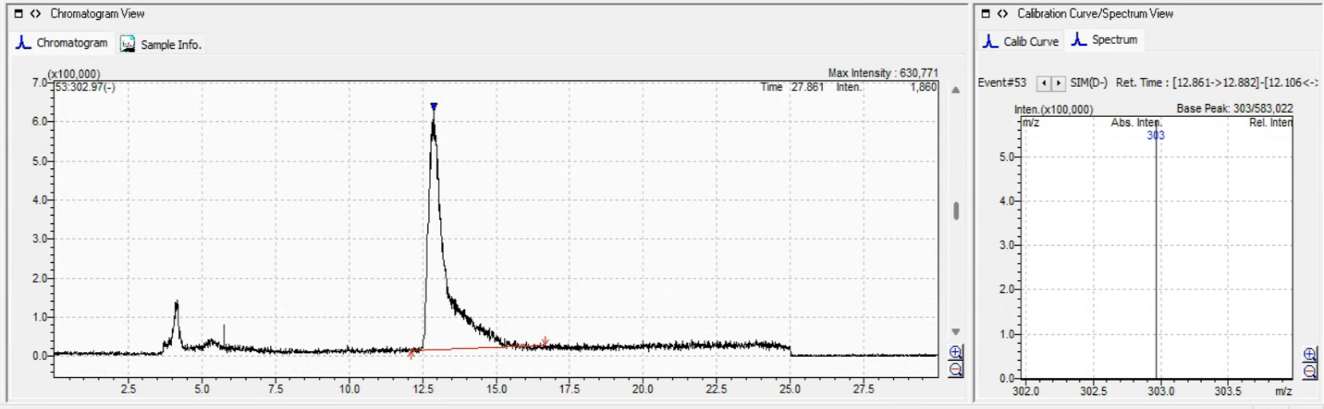


Fig. S 6. Representative chromatograms of ^13^C_7_-PFHpS (m/z 455.99). All analytes were measured in negative ion mode using Selected Ion Monitoring (SIM) on a Single Quadrupole LCMS-2050.

Sed-2 C3 (Fe(III) treatment) columns showed signal intensities of 21,901 to 44,583 for ^13^C_7_-PFHpS over the first pore volume after a 50-day incubation. Sed-2 C2 (NH_4_^+^ treatment) treatment had higher signal intensities for ^13^C_7_-PFHpS ranging from 56,334 to 103,985 over the first pore volume. Likewise, Sed-3 C3 (Fe(III) treatment) was the only treatment that had detectable ^13^C_4_-PFBA at an intensity range of 22,048 to 16,260 over the first pore volume.

When compared to the respective ^13^C standards, the retention time for the ^13^C_7_-PFHpS samples matched that of the standards at 19 ± 1 minutes and showed a total concentration of 4.2±1.2 ug/L of ^13^C_7_-PFHpS in the Sed-2 C3 (Fe(III) treatment) effluent and 9.6±2.3 ug/L of ^13^C_7_-PFHpS in the Sed-2 C2 (NH_4_^+^ treatment) effluent. For ^13^C_4_-PFBA, the retention time for both the standard and sample was 13±1 minute and showed a total concentration of 1 ug/L of ^13^C_7_-PFHpS in the Sed-3 C3 (Fe(III) treatment) effluent.

**References**

1. Shoemaker, J. and Tettenhorst, D. Method 537.1: Determination of Selected Per- and Polyfluorinated Alkyl Substances in Drinking Water by Solid Phase Extraction and Liquid Chromatography/Tandem Mass Spectrometry (LC/MS/MS). U.S. Environmental Protection Agency, Office of Research and Development, National Center for Environmental Assessment, Washington, DC, 2018.
2. Shimadzu. 2023. Analysis of 28 Common PFAS Compounds using Compact Single Quadrupole LCMS-2050. Application News SSI-LCMS-14. <https://www.ssi.shimadzu.com/sites/ssi.shimadzu.com/files/pim/pim_document_file/ssi/applications/application_note/22420/SSI-LCMS-149.pdf>
3. Huang, S., Smorada, C., Schaefer, C. E., Jaffe, P. R. 2024b. Stimulating *Acidimicrobium* sp. Strain A6 in iron-rich, acidic sediments from AFFF-impacted sites for PFAS defluorination. Sci. Total Environ. 955, 176801.
4. Chiavola, A., Marcantonio, C.D., Boni, M.R., Biagioli. S., Frugis, A., Cecchini, G. 2020.

Experimental investigation on the perfluorooctanoic and perfluorooctane sulfonic acids fate and behaviour in the activated sludge reactor Process. Saf. Environ. Prot., 134: 406-415.

1. Caporaso, J. G., Lauber, C.L., Walters, W.A., Berg-Lyons, D., Huntley, J., Fierer, N., Owens, S.M., Betley, J., Fraser, L., Bauer, M., Gormley, N., Gilbert, J.A., Smith, G., Knight, R. 2012. Ultra-high-throughput microbial community analysis on the Illumina HiSeq and MiSeq platforms, ISME J. 6.1621–1624.
2. Hach Company. (2017). Method 8043: Biochemical Oxygen Demand (BOD₅) in Water and Wastewater. Hach Company, Loveland, CO, USA.
3. Cao, L., Xu, W., Wan, Z., Li, G., Zhang, F. 2022. Occurrence of PFASs and its effect on soil bacteria at a fire-training area using PFOS-restricted aqueous film-forming foams. iScience, 25, 4, 104084.
4. Huang, S., Pilloni, G., Key, T., and Jaffé, P.R. 2024a. Defluorination of Various Perfluoro Alkyl Acids and Selected PFOA and PFOS Monomers by Acidimicrobium sp. Strain A6 Enrichment Cultures,” J. Hazard. Materials, Vol. 480, 2024, 136426.
5. Huang, S., Smorada, C., Schaefer, C. E., Jaffe, P. R. 2024b. Stimulating *Acidimicrobium* sp. Strain A6 in iron-rich, acidic sediments from AFFF-impacted sites for PFAS defluorination. Sci. Total Environ. 955, 176801.
6. Tang, Z., Song, X., Xu, M., Yao, J., Ali, M., Wang, Q., Zeng, J., Ding, S., Wang, C., Zhang, Z., Liu, X. 2022. Effects of co-occurrence of PFASs and chlorinated aliphatic hydrocarbons on microbial communities in groundwater: A field study. J. Hazard. Materials. 435. 128969.
7. Liu, H., Hu, W., Li, X., Hu, F., Liu, Y., Xiu, T., Liu, B., Xi, Y., Su, Z., Zhang, C. 2022. Effects of perfluoroalkyl substances on root and rhizosphere bacteria: Phytotoxicity, phyto-microbial remediation, risk assessment. Chemosphere. 289. 133137.
8. Tong, H., Hu, M., Li, F., Chen, M., Lv, Y. 2015, Burkholderiales participating in pentachlorophenol biodegradation in iron-reducing paddy soil as identified by stable isotope probing. Env Sci: Process & Impacts. 7
9. Wang, Y., Li, X., You, L., Hu, S., Fang, J., Hu, B., Chen, Z. 2024. Enhancement of PFAS stress tolerance and wastewater treatment efficiency by arbuscular mycorrhizal fungi in constructed wetlands. Env. Res. 263, 3, 120148.
